# Myostatin inhibition with orally administered *Lactobacillus casei* expressing a modified human myostatin protein: functional benefits and translational potential in advanced Duchenne muscular dystrophy

**DOI:** 10.3389/fneur.2025.1693484

**Published:** 2026-01-13

**Authors:** Jiwon Lee, Ju-A Kim, Yena Oh, Kwang Kim, Jeehun Lee

**Affiliations:** 1 Department of Pediatrics, Samsung Medical Center, Sungkyunkwan University School of Medicine, Seoul, Republic of Korea; 2 MOA Life Plus Co. Ltd., Yongin-si, Republic of Korea; 3 Future & Tech Corp., Daejeon, Republic of Korea

**Keywords:** Duchenne muscular dystrophy, myostatin inhibition, oral immunotherapy, *Lactobacillus casei*, aged *mdx* mouse model, pgsA-based antigen display

## Abstract

**Background:**

Duchenne muscular dystrophy (DMD) is a progressive neuromuscular disorder that requires novel therapeutic approaches beyond dystrophin restoration. Myostatin, a negative regulator of muscle growth, has emerged as a promising target to enhance muscle mass and function.

**Methods:**

We evaluated the efficacy of an orally administered *Lactobacillus casei* strain expressing a modified human myostatin protein (BLS-M22), in 32-week-old *mdx* mice. Animals received BLS-M22 or control (*L. casei*-pgsA) for 8 weeks (control group = 8, treated group = 7) and 12 weeks (control group = 8, treated group = 9).

**Results:**

BLS-M22 elicited a robust systemic anti-myostatin antibody response and significantly reduced serum creatine kinase levels, indicating attenuated muscle damage. Treated mice showed improved endurance in rotarod performance. However, no significant differences were observed in body weight, muscle fiber cross-sectional area, or fibrosis, reflecting the limited regenerative capacity at an advanced disease stage.

**Conclusion:**

This study demonstrates that myostatin inhibition with orally administered *L. casei* expressing a modified human myostatin protein confers functional benefits even in advanced DMD, while highlighting its therapeutic limitations without concomitant dystrophin restoration. As a cost-effective, non-invasive, and immunologically distinct platform, this system holds translational potential not only for DMD but also for broader applications in sarcopenia and metabolic disorders.

## Introduction

Duchenne muscular dystrophy (DMD) is a severe X-linked neuromuscular disorder caused by mutations in the dystrophin gene that lead to progressive muscle degeneration, loss of ambulation, and premature mortality (1–3). Natural history studies show that most patients lose independent ambulation in early adolescence and experience respiratory insufficiency in their twenties (2, 4).

Current standards of care, including corticosteroids, have demonstrated efficacy in extending the ambulatory period, reducing severity of scoliosis, and preserving pulmonary function (5, 6). However, disease progression remains inevitable, and therapeutic options are particularly limited for patients in advanced and non-ambulatory stages (5, 7). In advanced disease stage corresponding to the non-ambulatory phase, supportive measures such as respiratory assistance, rehabilitation, and cardiac care become critical; however, they do not prevent further functional decline (5–7). These limitations underscore the urgent need for adjunctive therapies aimed at preserving residual muscle function and enhancing quality of life in the later stages of the disease.

Myostatin, a member of the transforming growth factor-beta (TGF-β) superfamily, functions as a potent negative regulator of skeletal muscle growth (8, 9). Loss-of-function mutations in MSTN, the gene encoding myostatin, lead to significant muscle hypertrophy, making it an attractive therapeutic target for muscle-wasting conditions (9–12). Although various pharmacological myostatin inhibitors, such as monoclonal antibodies and antisense oligonucleotides, have been evaluated in clinical and preclinical studies, their success has been variable, particularly in advanced disease status, where both muscle mass and myostatin levels are diminished (13–17).

We employed aged *mdx* mice (≥32 weeks) because they represent the preclinical model of dystrophin deficiency and, at this age, recapitulate key features of advanced, non-ambulatory–stage DMD—including increased fibrosis, reduced myofiber number, and limited regenerative capacity. This model permits controlled oral dosing of BLS-M22 and serial assessments of rotarod performance, serum CK, and anti-myostatin IgG, together with histo-pathology and immune profiling that are impractical in patients at this stage. Establishing efficacy and safety in this advanced disease-relevant model is a necessary step toward clinical translation.

We previously reported that oral administration of a genetically modified *Lactobacillus casei* expressing human myostatin (BLS-M22), successfully enhanced mucosal and systemic immune responses in young *mdx* mice, resulting in improved motor performance and muscle pathology in DMD (18). This approach utilizes a bacterial delivery system to stimulate anti-myostatin immunity through gut-associated lymphoid tissue, offering a non-invasive and potentially scalable therapeutic strategy (19–21).

This study aimed to evaluate the therapeutic potential of BLS-M22 in aged *mdx* mice, a preclinical model of advanced-stage DMD, by investigating its capacity to induce systemic immunity, improve muscle function, and attenuate muscle damage. Moreover, we explored the feasibility of applying this platform as an adjunctive option in advanced disease stages, where conventional therapies offer only limited efficacy.

## Materials and methods

### Bacterial strains, cloning, and construction of surface-expressing plasmids

This study utilized the previously described BLS-M22 strain (18). To express antigens on the surface of *L. casei*, a pKV vector containing a highly constitutive promoter (the aldolase promoter derived from *L. casei*) and pgsA (from *Bacillus subtilis*) was used as the anchoring domain. The modified myostatin sequence comprises four repeats of Myo2L, four linker sequences, and a mature myostatin domain.

Myo2L is an oligopeptide sequence representing amino acid residues 316th–330th of human pro-myostatin (*Homo sapiens*; GenBank accession number: ABI48422.1), which was selected for its high antigenicity. DNA fragment encoding the modified myostatin was chemically synthesized, optimized for codon usage in *L. casei*, and confirmed by sequencing (Cosmo Genetech, Seoul, Korea).

A recombinant plasmid was constructed by inserting a modified myostatin sequence into the C-terminal region of the pgsA sequence in the pKV vector. This was achieved using BamHI and XbaI restriction enzymes. The resulting expression vector encoded the pgsA-modified myostatin fusion protein.

The constructed vectors were initially propagated in *Escherichia coli* DH5α cells and subsequently introduced into *L. casei* via electroporation. Recombinant *L. casei* strains were cultured in De Man, Rogosa, and Sharpe (MRS) medium supplemented with erythromycin (16 μg/mL) at 30 °C. For propagation in *E. coli*, the DH5α strain was grown in Luria–Bertani (LB) medium containing erythromycin (150 μg/mL) at 37 °C.

### Animals

This study was conducted in accordance with ethical guidelines and approved by the Institutional Animal Care and Use Committee of the Samsung Biomedical Research Institute (SBRI; approval number: C-B1-309-1). The SBRI is an internationally accredited facility certified by the Association for Assessment and Accreditation of Laboratory Animal Care International and adheres to the Institute of Laboratory Animal Resources guidelines.

Male and female *mdx* mice (C57BL/10ScSn-*Dmd^mdx^*/J) were obtained from the Jackson Laboratory (Bar Harbor, ME, United States). Male *mdx* mice were selected for the study, and their transgenic status was confirmed via genotyping following the protocol recommended by the Jackson Laboratory (Protocol 21,940). To maintain consistency and reduce variability, only male mice were used in all experimental procedures.

Mice were maintained in a specific pathogen-free (SPF) facility in individually ventilated cages (≤5 mice/cage) with corncob bedding, nesting material, and environmental enrichment (plastic shelters/tunnels). The animal room was controlled at 22 ± 2 °C, 40–60% humidity, with a 12:12-h light/dark cycle (lights on 07:00–19:00). Animals had ad libitum access to autoclaved standard chow and filtered water. A 7-day acclimatization period preceded any procedure.

Health was checked daily by trained staff; body weight and general condition were recorded weekly (and at experimental time points). All efforts were made to minimize pain and distress: procedures were performed during the light phase; brief handling was used; and anesthesia was provided for blood collection and terminal procedures (ketamine/xylazine, dose per protocol). Humane endpoints (≥20% weight loss, severe lethargy, dyspnea, or ulcerative dermatitis) were predefined; any animal meeting criteria was euthanized. At study end, animals were euthanized under deep anesthesia followed by cardiac exsanguination and cervical dislocation, in accordance with institutional policy. Cage changes and sanitation followed standard schedules; room entry and handling were performed by personnel blinded to treatment whenever feasible. Randomization procedures and group sizes are described in the Animals section; no unplanned adverse events occurred during housing or handling.

**Figure 1:**
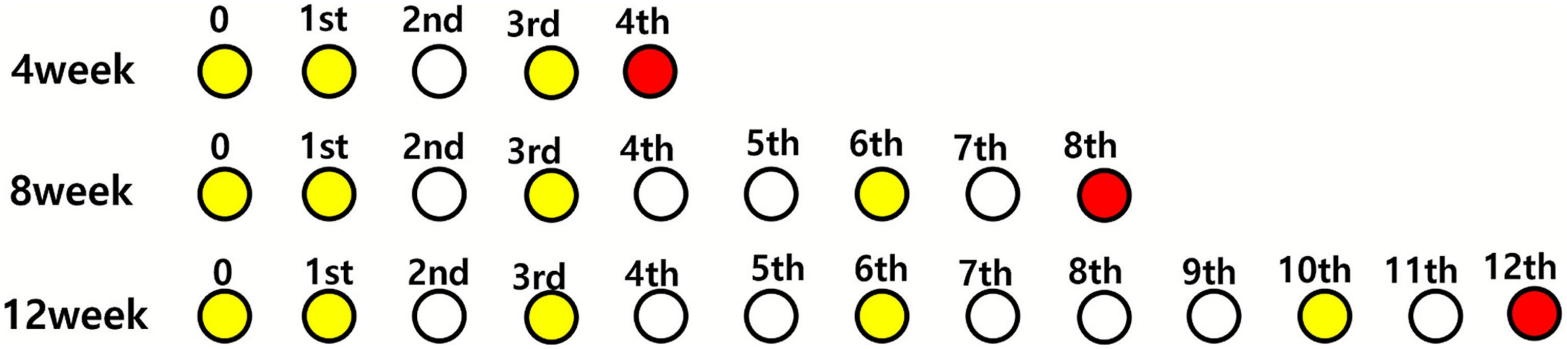
Dosing schedule of investigational products. Experimental timeline for 32-week-old male *mdx* mice. Animals were fed powder-formulated diets containing either *Lactobacillus casei*-pgsA presenting modified human myostatin (BLS-M22, treatment group) or *L. casei*-pgsA alone (control group). Dosing was performed daily for the first 2 weeks, followed by booster administrations at scheduled intervals for 8 or 12 weeks. Yellow circles indicate dosing weeks; red circles indicate sacrifice time points.

At the start of the study, the experimental animals were 32 weeks old. The animals were randomized into groups as shown in Figure 1. The group allocations and sample sizes were as follows: eight untreated (control) and seven treated mice in the 8-week group, eight untreated (control) and nine treated mice in the 12-week group, and four control and two treated mice in the 4-week group. The 4-week group was included solely to assess the timing of anti-myostatin antibody generation. However, the limited sample size precluded its inclusion in the statistical analysis to ensure data robustness.

### Preparation and administration of investigational products

Investigational products were prepared as described previously (18). For the treatment group, *L. casei*-pgsA presenting modified human myostatin (BLS-M22) was used, while the control group received *L. casei*-pgsA without surface myostatin display. Both investigational products were prepared as heat-inactivated and lyophilized powders for oral administration.

The seed culture of investigational products was grown in MRS broth supplemented with erythromycin (16 μg/mL) at 30 °C for 18 h in a 1.5 L fermenter, reaching an optical density (OD) of 6.0 ± 1.0 at 600 nm (OD600). The primary culture was then transferred to a 50 L fermenter and grown for an additional 18–20 h until reaching an OD600 of 9.0 ± 1.0. After cooling the culture medium to 10 °C, maltose was added to a final concentration of 5% and incubated for 2 h to stabilize the cells. Gradual heat inactivation was performed by sequentially increasing the temperature to 60 °C, 80 °C, 85 °C, and finally 90 °C, with cooling steps at 30 °C between temperature increments. This ensured complete inactivation while preserving the integrity of the surface antigens.

The inactivated cells were collected by centrifugation at 12,000 × g at 4 °C for 9 min, washed three times with sterile distilled water, and freeze-dried. The lyophilized powder was stored in sterile aluminum bags at 4 °C, with a final concentration of 1.2 × 10 colony-forming units (CFU) per gram.

The investigational products were administered using a mixed-with-feed method, with 3% BLS-M22 or *L. casei-pgsA* added to standard mouse feed. The initial doses were administered daily for the first 2 weeks, followed by booster doses at specific intervals, as outlined in Figure 1.

### Physical measurement and behavioral evaluation

Body weight was measured weekly throughout the experimental period and at euthanasia. Behavioral evaluation of motor function was conducted using a rotarod apparatus (Samkwang, Korea). The apparatus was set to rotation speeds of 5 and 10 rpm to evaluate the functional capabilities of the mice. Each mouse underwent six training sessions daily for three consecutive days during the week before sacrifice to ensure familiarity with the test. For the evaluation, each mouse performed three trials, and the maximum value among the three trials was recorded as the final result.

### Blood collection

Blood samples were collected after euthanasia under general anesthesia. Mice were anesthetized via intraperitoneal injection of ketamine (0.1–0.5 mg/kg) until full sedation was achieved. Once anesthetized, whole blood was collected via direct puncture of the left ventricle using a sterile syringe. The collected blood samples were centrifuged at 2,000 × g for 5 min to separate the serum, which was stored at −70 °C for downstream analyses, including quantification of anti-myostatin IgG and serum creatine kinase (CK) levels.

### Serum CK assay

Serum CK levels were measured using an indirect colorimetric assay kit (Sig-ma-Aldrich, St. Louis, MO, United States). The assay was performed according to the manufacturer’s instructions, using the standards provided to ensure accuracy and consistency.

### Quantitation of anti-myostatin IgG antibody

Serum anti-myostatin IgG antibody levels were quantified using an ELISA-based protocol as previously described (18). Recombinant myostatin (R&D Systems, Minneapolis, MN, United States) was used to coat 96-well plates, and antibody binding was detected using Horseradish Peroxidase -conjugated secondary antibodies. Optical density was measured at 450 nm using a microplate reader (Bio-Rad, Hercules, CA, United States).

### Tissue harvest and histological analysis

Muscle tissues, including the gastrocnemius and extensor digitorum longus (EDL), were harvested following cardiac perfusion with normal saline to minimize blood contamination and fixed in 10% neutral-buffered formalin. Fixed tissues were embedded in paraffin blocks, and 4 μm-thick sections were prepared for staining and analysis.

Hematoxylin and eosin (H&E) staining was used to evaluate the general muscle histology, including fiber morphology, cellular infiltration, and structural abnormalities. Digital images of stained sections were captured using an Olympus IX51 inverted micro-scope (Tokyo, Japan).

For fibrosis analysis, sections were stained using Masson’s Trichrome Stain Kit (Sigma-Aldrich, St. Louis, MO, United States). Fibrotic areas were visualized as blue-stained regions, and the percentage of fibrosis-positive areas was quantified using the ImageJ soft-ware (version 1.51, National Institutes of Health, Bethesda, MD, United States).

### Statistical analysis

Statistical analyses were performed using the Prism software (version 10.2.3; GraphPad Software, San Diego, CA, United States). Data distribution was assessed using the Shapiro–Wilk test. For variables that followed a normal distribution (e.g., muscle fiber cross-sectional area and fibrosis area), statistical comparisons between groups were conducted using an unpaired *t*-test. For other variables in which at least one group failed the normality test (e.g., CK levels, rotarod performance, body weight, and IgG titers), the non-parametric Mann–Whitney *U* test was applied. Differences with *p* < 0.05 were considered statistically significant.

## Results

Descriptive data comparing the control and treatment groups at the 8^th^ and 12^th^ weeks are presented in Table 1. All the mice were well maintained there was no drug related adverse effects. As study went on, the movements of the mice markedly decreased.

**Table 1:** Difference in variables between control and treated groups.

Variable	Dosing week	Control group (median, IQR)	Treated group (median, IQR)	^#^*p*-value
Number	4th week	4	2	
	8th week	8	7	
	12th week	8	9	
Body weight (gram)	4th week	36.5 (34.3–38.8)	32 and 38	NA
	8^th^ week	36.5 (34.5–39.0)	34.0 (32.0–36.0)	0.05
	12th week	34.0 (34.0–39.5)	35.0 (33.5–36.0)	0.98
Serum CK, (IU/L)	4th week	9,850 (6,300–10,200)	6,700 and 11,400	NA
	8th week	14,000 (12,125–19,775)	9,600 (8,800–11,400)	< 0.01
	12th week	16,750 (8,650–25,600)	9,700 (6,500–12, 950)	0.10
Antimyostatin Ab (OD)	4th week	0.16 (0.16–0.18)	0.33 and 0.36	NA
	8th week	0.16 (0.15–0.17)	0.58 (0.51–0.67)	< 0.001
	12th week	0.15 (0.13–0.16)	0.50 (0.45–0.56)	< 0.001
Rotarod, 5 rpm (sec)	4th week	18.5 (7.0–233)	60 and 200	NA
	8th week	16.5 (10.8–27.3)	18.0 (15.0–121)	0.45
	12th week	21.0 (11.3–48.0)	300 (217–300)	< 0.001
Rotarod, 10 rpm (sec)	4th week	12.5 (10–18.8)	36 and 30	NA
	8th week	7.5 (6.0–33.8)	69.0 (44.0–92.0)	0.01
	12th week	8.00 (4.5–46.0)	110 (74.0–191)	< 0.001
Cell surface area, gastrocnemius	8th week	843.8 ± 75.6 (*n* = 32)	934.8 ± 74.9 (*n* = 40)	0.40
	12th week	793.1 ± 71.0 (*n* = 32)	878.5 ± 70.4 (*n* = 40)	0.40
Cell surface area, extensor digitorum longus	8th week	519.5 ± 37.8 (*n* = 52)	590.6 ± 31.7 (*n* = 52)	0.15
	12th week	507.2 ± 30.4 (*n* = 73)	561.2 ± 25.4 (*n* = 72)	0.18
Fibrosis area, gastrocnemius	8th week	22.5 (19.0–28.3)	22.0 (16.0–25.0)	0.51
	12th week	19.0 (15.3–29.5)	22.5 (19.8–25.8)	0.68
Fibrosis area, extensor digitorum longus	8th week	10.0 (7.25–12.8)	11.0 (5.00–15.0)	0.74
	12th week	8.5 (6.25–12.3)	9.0 (5.5–12.0)	1.0

IQR, interquartile range; CK, Creatine kinase; OD, optical density; rpm, revolutions per minute; NA, not assessed.

^#^*p*-value, unpaired *t*-test.

### Systemic immunogenicity: production of anti-myostatin IgG antibodies

**Figure 2:**
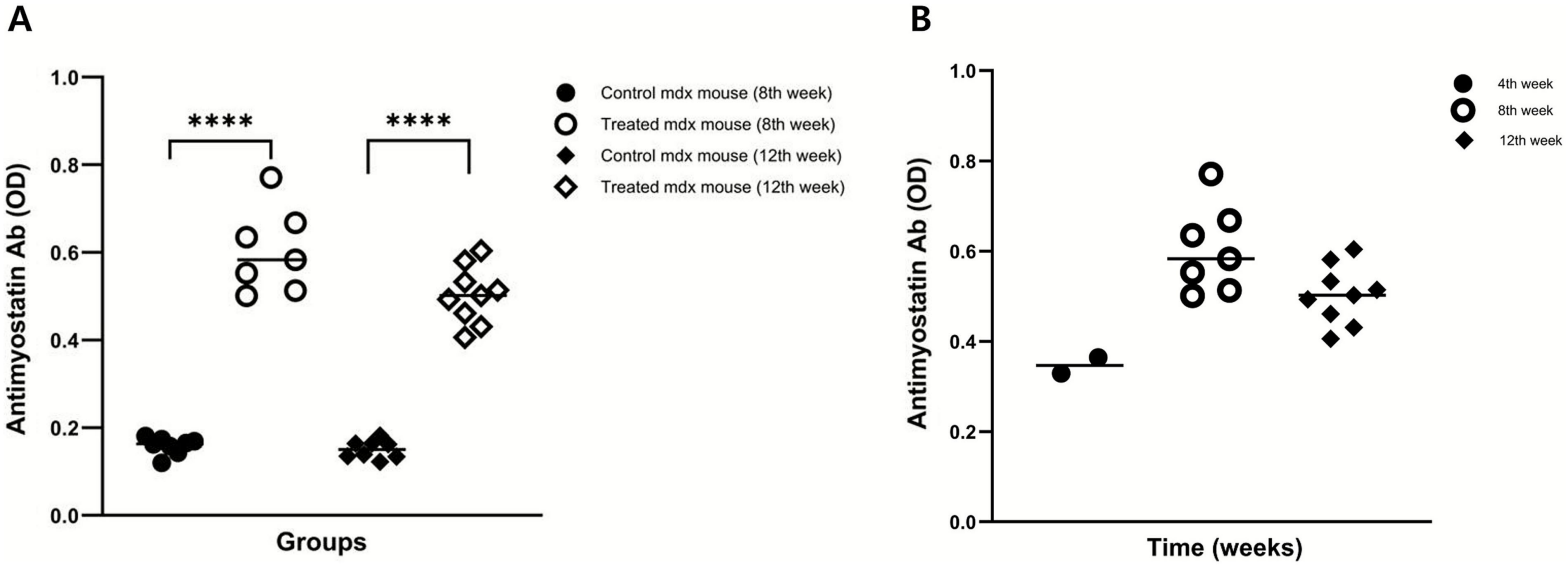
Induction of serum anti-myostatin IgG antibodies. **(A)** Serum anti-myostatin IgG levels were significantly higher in the treatment group than in the control group at weeks 8 and 12. **(B)** Serial measurements at weeks 4 (*n* = 2), 8 (*n* = 7), and 12 (*n* = 9) demonstrated a sustained antibody response over time. *p* < 0.0001.

To assess systemic immunogenicity, the serum levels of anti-myostatin IgG were measured. Treatment group exhibited significantly higher antibody titers than control group at both 8 and 12 weeks. Optical density (OD) values were as follows: 8 weeks – 0.60 (IQR: 0.58–0.64) in the treatment group vs. 0.16 (IQR: 0.15–0.17) in the control group (*p* < 0.0001); 12 weeks – 0.50 (IQR: 0.49–0.52) vs. 0.15 (IQR: 0.14–0.16), respectively (*p* < 0.0001; Table 1 and Figure 2A). Serial measurements of anti-myostatin IgG antibody titers at weeks 4 (*n* = 2), 8 (*n* = 7), and 12 (*n* = 9) demonstrated a sustained antibody response over time, peaking at the 8th week (Figure 2B).

### Reduction in serum CK levels

To evaluate the protective effects of investigational product (BLS-M22) against muscle damage, serum CK levels were measured. In the control group, CK levels increased over time, reaching 14,000 IU/L (IQR: 12,125–19,775 IU/L) at 8 weeks and 16,750 IU/L (IQR: 8,650–25,600 IU/L) at 12 weeks.

**Figure 3:**
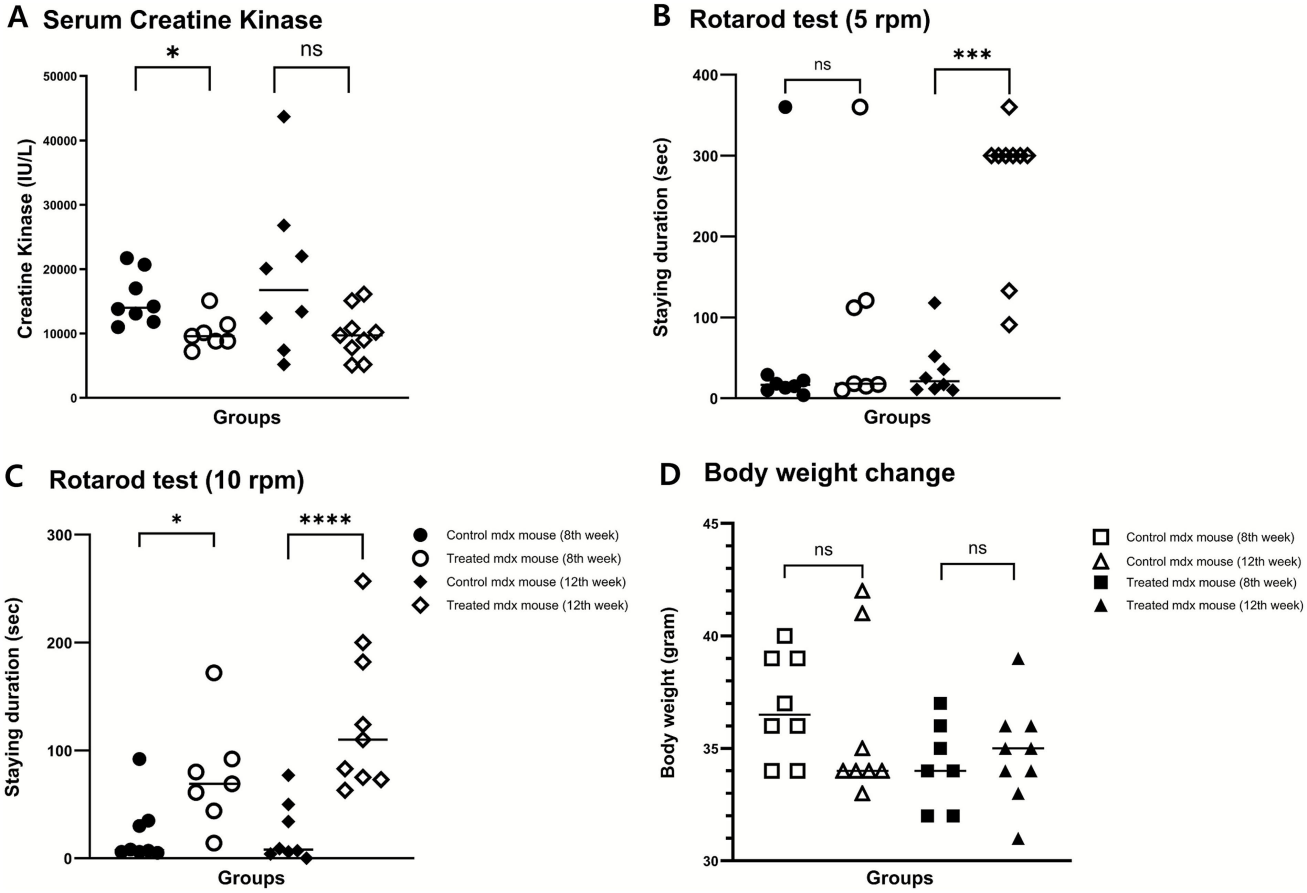
Protective and functional effects of investigational products. **(A)** Serum creatine kinase (CK) levels were significantly lower in the treatment group at week 8 (*p* < 0.01) and remained lower at week 12 (*p* = 0.06). **(B)** Rotarod performance at 5 rpm showed a trend toward improvement at week 8 and a significant increase in latency to fall at week 12 (*p* < 0.001). **(C)** At 10 rpm, treatment mice performed significantly better than controls at both week 8 (*p* < 0.05) and week 12 (*p* < 0.001). **(D)** Body weight showed divergent trends: controls exhibited a slight decline from week 8 to 12, whereas treated mice maintained a mild increase (both not significant). *p < 0.05; **p < 0.01; ***p < 0.001; ns, not significant.

In contrast, treatment group exhibited lower CK levels at both times. At 8 weeks, CK levels were significantly reduced in the treatment group (9,600 IU/L, IQR: 8,800–11,400 IU/L; *p* < 0.01). At 12 weeks, CK levels remained lower in the treatment group (9,700 IU/L, IQR: 6,500–12,950) than in the control group, although the difference was not statistically significant (*p* = 0.10). This trend suggested a sustained protective effect against muscle degradation (Figure 3A).

### Effects on physical activity and body weight

Motor function, as evaluated using the rotarod test, improved in the treatment group. At 5 rpm, the treatment group showed a trend toward improvement at week 8 (*p* = 0.45) and a statistically significant enhancement at week 12 (*p* < 0.001). At 10 rpm, the treatment group exhibited significantly better performance at both 8 weeks (*p* = 0.01) and 12 weeks (*p* < 0.001), indicating enhanced motor coordination and endurance (Figures 3B,C).

The control group showed a decreasing trend in body weight between weeks 8 and 12 (*p* = 0.29), whereas the treatment group showed a slightly increasing trend over the same period (*p* = 0.74; Figure 3D).

### Histological analysis: muscle fiber size and fibrosis

**Figure 4:**
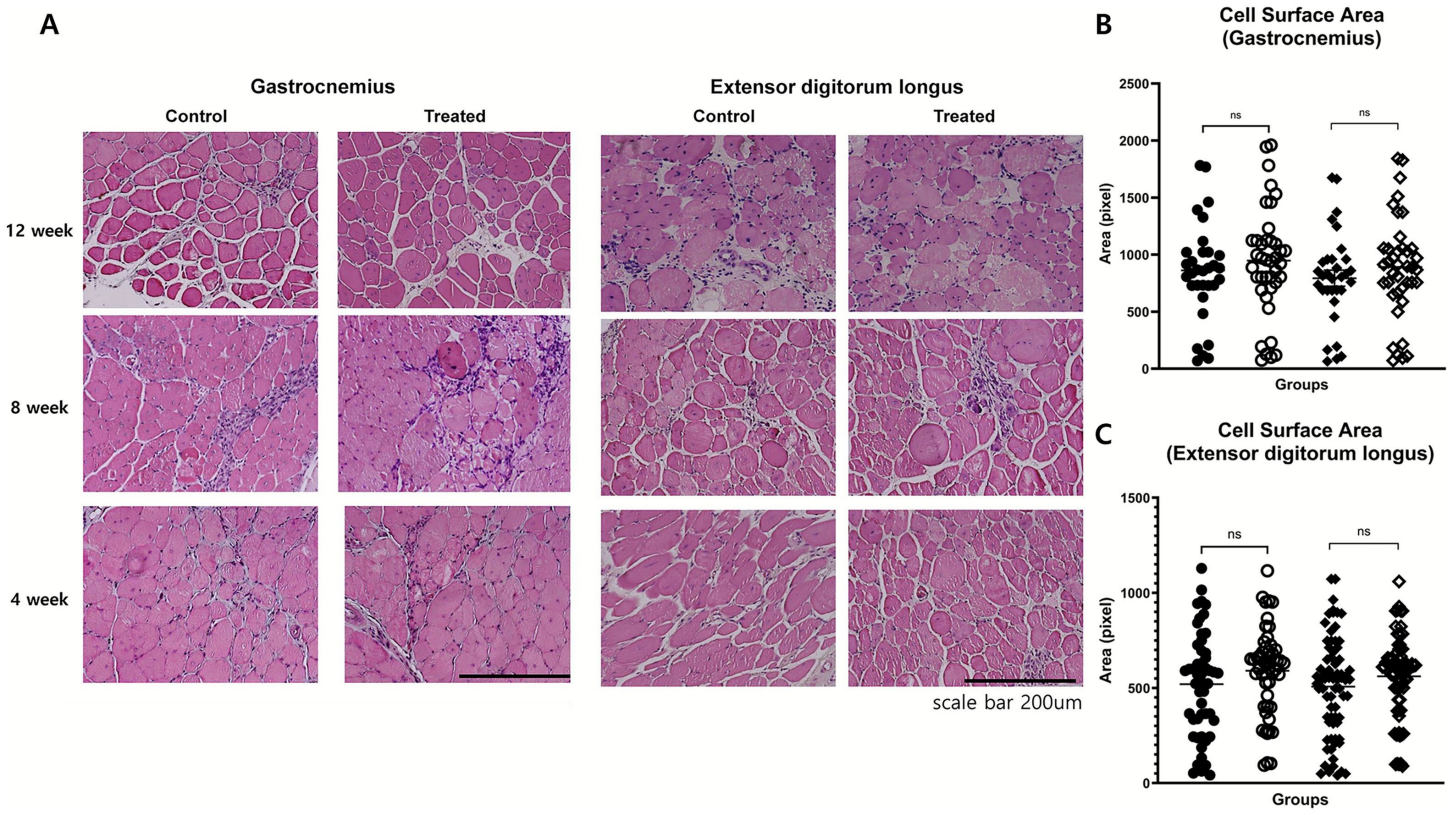
Histological assessment of muscle morphology. **(A)** Representative H&E-stained cross-sections of gastrocnemius and extensor digitorum longus (EDL) muscles at weeks 4, 8, and 12 in control and treatment groups. No marked differences were observed in fiber size variability, central nucleation, or inflammatory infiltration. **(B,C)** Quantitative analysis of fiber cross-sectional area in gastrocnemius **(B)** and EDL **(C)** muscles at weeks 8 and 12 showed no significant group differences.

Histological analyses of the EDL and gastrocnemius muscles were performed to evaluate morphological changes. The muscle fiber cross-sectional area was assessed using H&E staining. There was no significant difference in cross-sectional area between the treatment group and control groups in either the gastrocnemius or extensor digitorum longus muscles. This indicates that investigational product treatment did not induce measurable muscle fiber hypertrophy in these muscle groups at 8 or 12 weeks. The consistent muscle fiber size across treatment conditions and time points reflects limited histological improvement in aged *mdx* mice, likely due to the reduced regenerative capacity associated with advanced disease (Figure 4).

**Figure 5:**
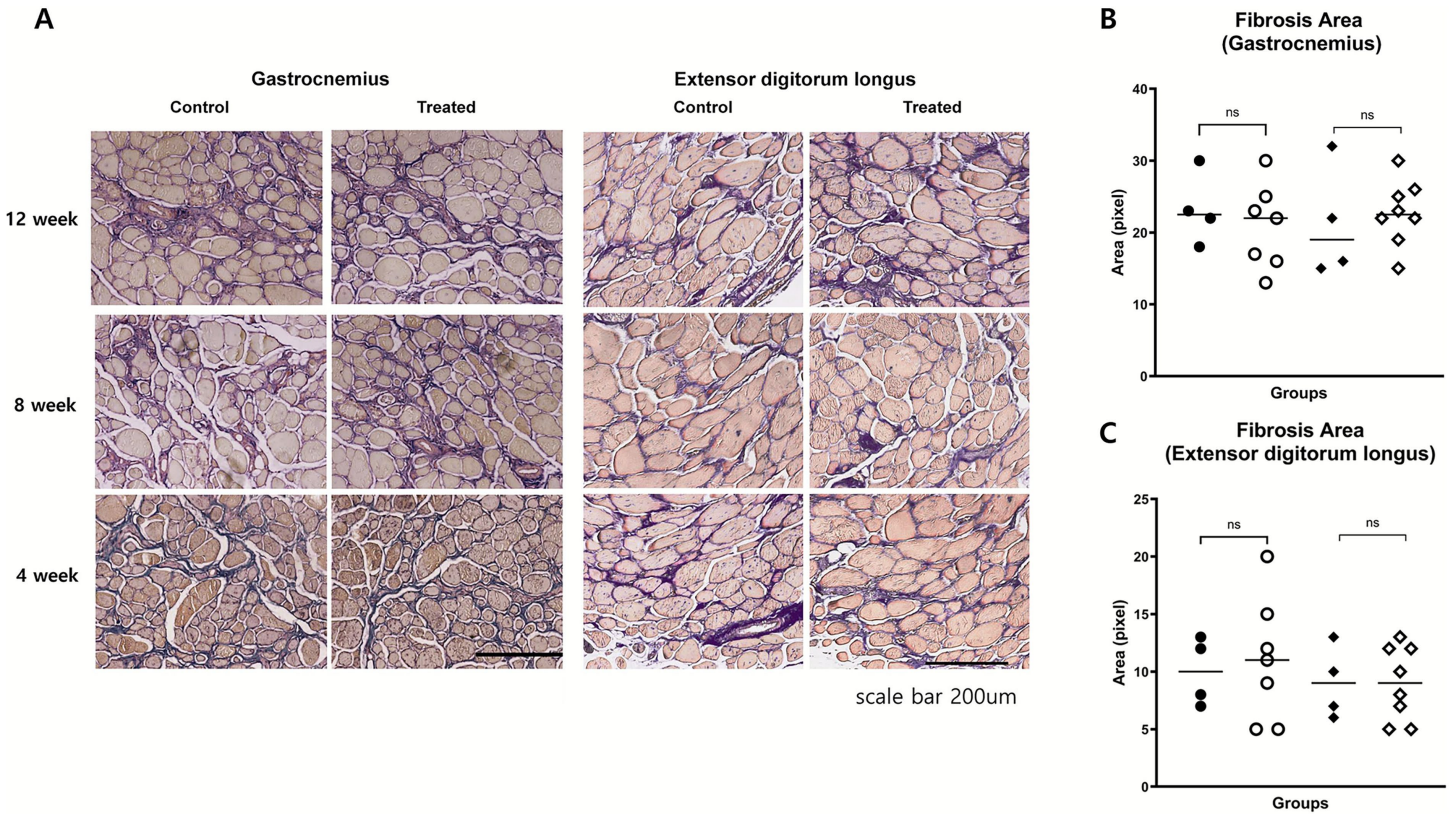
Histological assessment of muscle fibrosis. **(A)** Representative Masson’s trichrome-stained images of gastrocnemius and EDL muscles at weeks 4, 8, and 12. Fibrotic areas (blue) appeared qualitatively similar between groups. **(B,C)** Quantitative analysis of fibrosis area fraction in gastrocnemius **(B)** and EDL **(C)** muscles at weeks 8 and 12 showed no significant differences between groups.

Fibrosis was evaluated using Masson’s trichrome staining. In the extensor digitorum longus, the fibrosis area fraction was comparable between the groups at 8 (*p* = 0.74) and 12 weeks (*p* = 1.0). In the gastrocnemius muscle, no significant differences were noted at 8 weeks (*p* = 0.51) or 12 weeks (*p* = 0.68; Figure 5). The typical histopathological features of DMD were also evaluated to assess potential treatment-related changes. These included variability in muscle fiber size, the presence of centrally located nuclei, and the extent of inflammatory cell infiltration. Across all these parameters, no notable differences were observed between the treatment group and the control group (Figure 4). These findings suggest that, investigational product did not induce measurable histological improvements in these characteristic pathological features of dystrophic muscle.

## Discussion

This study evaluated the therapeutic potential of an *L. casei* strain engineered to express a modified human myostatin protein, in aged *mdx* mice, a model of advanced-stage DMD. The investigational product effectively induced circulating anti-myostatin IgG antibodies and conferred measurable functional benefits, including improved rotarod performance and reduced serum CK levels. Although no statistically significant differences in body weight were observed, the treatment group tended to maintain weight over time, in contrast to the control group, which showed a gradual decline. Muscle histopathological parameters such as fiber size and fibrosis also remained unchanged. Although the therapeutic effects were more modest than those observed in young *mdx* mice, these findings support the potential role of investigational product as an adjunct therapy for advanced DMD, particularly when combined with dystrophin-restoring approaches.

In this study, investigational product (BLS-M22) demonstrated its ability to induce the production of circulating anti-myostatin antibodies by presenting modified myostatin proteins on the surface of *L. casei* as same as our previous study (18). In our previous studies, it enhanced both mucosal IgA and systemic anti-myostatin IgG antibodies, effectively reducing serum myostatin levels in young *mdx* mice. This immune response to a self-antigen, such as myostatin, is atypical and highlights the unique design of the investigational product. The modified myostatin protein used in BLS-M22 elicits an immune response against native myostatin through a strategic design. By incorporating four repeats of the myostatin active domain segment fused to pgsA, it ensured the effective display of the antigen on the bacterial surface. The identical amino acid sequences of the antigenic myostatin moiety in BLS-M22 and human myostatin likely enhance its immunogenicity (18). This vaccination strategy primarily initiates mucosal immunity followed by systemic immunity, leveraging the ability of the immune system to recognize extracellularly accessible antigens more effectively (19, 20).

Serum CK is a well-established biomarker for the diagnosis of DMD (22). Although CK levels do not directly reflect ongoing disease activity, comparisons between control and treatment groups can indicate the degree of muscle degradation. A reduction in serum CK levels reflects the protective effect of myostatin inhibition by BLS-M22. In the control group, mean serum CK levels were 14,000 IU/L at 8 weeks and 16,750 IU/L at 12 weeks. These values in aged mdx mice were relatively lower than those previously observed in young mdx mice (21,257 IU/L and 48,160 IU/L, respectively) in our earlier study. This age-related difference parallels findings in humans, where serum CK levels decline with age, reflecting reduced residual muscle mass (22, 23). In the present study, a significant reduction was observed in the treatment group at 8 weeks compared with controls. By 12 weeks, however, the marked variability in serum CK levels obscured between-group differences (Figure 3), underscoring the need for replication in future trials.

The rotarod test demonstrated improved motor function in the treatment group, particularly at the higher velocity of 10 rpm. While no consistent improvements were observed at 8 weeks, a clear enhancement was evident at 12 weeks at both velocities. These findings indicate that the therapeutic effect on motor performance becomes more apparent as disease severity increases. Although significant differences were observed between groups, the rotarod was conducted at a relatively low speed. Standard protocols typically employ initial speeds ranging from 5 to 45 rpm with gradual acceleration, depending on the functional capacity of the mouse (24). In *mdx* mice, similar starting speeds are recommended, with a standard test duration of 500 s. The aged *mdx* mice in this study exhibited reduced tolerance compared with younger *mdx* mice reported previously (25). Taken together, these results suggest that myostatin inhibition with BLS-M22 confers measurable, albeit limited, functional benefits even in advanced stages of disease. Importantly, although patients with DMD typically receive corticosteroids from an early age along with multidisciplinary care such as rehabilitation, respiratory support, and cardiac monitoring, the disease nonetheless progresses gradually. In this context, the observed improvements in aged *mdx* mice highlight the potential of BLS-M22 to provide additional functional benefit beyond current standards of care, supporting its investigation as a complementary therapeutic strategy in both early and later stages of disease.

There was no significant difference in body weight between the two groups. Although not statistically significant, the treatment group tended to maintain slightly higher body weight over time, whereas the control group exhibited a gradual decline. These findings suggest that myostatin inhibition with BLS-M22 was insufficient to elicit a meaningful increase in muscle mass at an advanced stage of DMD. Because precise matching of baseline body weights at 32 weeks of age was challenging, longitudinal changes relative to controls provide a more reliable assessment. The limited efficacy observed in aged mice, compared with younger models, likely reflects the advanced disease stage, which restricts functional restoration. In aged *mdx* mice, severe muscle loss reduces the available tissue for hypertrophy in response to myostatin inhibition. While residual fibers may undergo hypertrophy, the overall reduction in fiber number at late stages of disease ultimately constrains the extent of functional improvement (26–28).

In this study, the persistent pathological features of the DMD muscle – including hypertrophy, atrophy, internal nuclei, inflammatory infiltration, and fibrosis – were not reversed by myostatin inhibition. By contrast, previous studies in younger *mdx* mice demonstrated that BLS-M22 improved muscle pathology by increasing the cross-sectional area and reducing fibrosis (18). This discrepancy is likely attributable to the advanced disease stage of aged *mdx* mice, in which regenerative capacity is severely diminished owing to satellite cell exhaustion, chronic inflammation, and fibrotic remodeling. Nevertheless, the observed functional and biochemical improvements imply that myostatin inhibition may confer benefits beyond structural regeneration. One possible mechanism is reduced sarcolemma fragility, leading to decreased muscle enzyme leakage and enhanced endurance. This hypothesis is supported by prior evidence that myostatin signaling influences cytoskeletal stability and membrane repair processes (29). Furthermore, although myostatin inhibition can downregulate profibrotic pathways such as TGF-β signaling, its impact is likely limited in tissues with established fibrosis and cellular senescence (30). Thus, while BLS-M22 alone may not reverse chronic fibrotic remodeling, it may still attenuate ongoing damage and preserve residual function.

In addition, the efficacy of myostatin inhibition may be constrained by reduced myostatin expression in advanced neuromuscular disorders. In conditions such as spinal muscular atrophy and late-stage DMD, circulating myostatin levels decline markedly, reducing the availability of molecular targets for inhibition (31). This diminished target landscape further compromises therapeutic efficacy, particularly in severely atrophic muscles, where both structural integrity and molecular substrates for regeneration are profoundly impaired.

Myostatin, a member of the TGF-β family, is a key negative regulator of muscle growth, and its inhibition has emerged as a promising therapeutic approach for muscle atrophy (8, 9, 32, 33). Two major strategies have been developed: monoclonal antibodies (e.g., MYO-029, BMS-986089, domagrozumab, ACE-083/−031, BYM338, and bimagrumab) and antisense oligonucleotides (14, 29, 34). Although these approaches demonstrated preclinical efficacy, clinical trials have generally failed to show robust improvements in motor strength (14, 15, 35, 36). An anti-myostatin adnectin, taldefgrobep alfa was tried in ambulatory boys with DMD-a phase 1b/2 trial assessing safety (NCT02515669) and a phase 2/3 trial (NCT03039686). The result showed good tolerability in healthy adult volunteers. It did not meet the primary endpoint analysis threshold of change from baseline of ≥1.5 points on North Star Ambulatory Assessment total score (37).

Combination approaches have also been investigated, particularly antisense oligonucleotides targeting both dystrophin and myostatin, which yielded superior outcomes to dystrophin targeting alone in preclinical models (38–40). Importantly, the magnitude of benefit varied by disease stage, with neonatal mdx mice showing clear histological improvement, while aged mice exhibited only modest effects. These observations highlight the synergistic potential of combined therapy and the importance of early intervention. Taldefgrobep alfa in participants with 5q autosomal recessive SMA who are on a stable regimen of nusinersen and/or risdiplam and/or who have a history of receiving onasemnogene abeparvovec, regardless of their SMA type or ambulatory status has been tried in phase 3, randomized, double-blind, placebo-controlled designed study (41).

BLS-M22 represents a novel oral strategy for myostatin inhibition, distinguished by its mucosal immune activation and the advantages of cost, convenience, and reversibility. Although promising, direct comparative studies are needed to establish its efficacy and safety relative to existing myostatin-targeting agents. Beyond DMD, myostatin inhibition has also been explored in sarcopenia, cancer cachexia, and metabolic disorders (13, 14, 42). Clinical and preclinical studies consistently suggest that myostatin inhibition can enhance muscle mass, improve physical performance, and favorably influence metabolic outcomes, underscoring its broad therapeutic potential.

A first-in-human study in healthy adults has been initiated, and results are not yet available (16). Pending those data, an initial patient study may be considered. Given that myostatin blockade alone has yielded limited functional benefit in advanced DMD (34), we anticipate BLS-M22 will be best positioned as an adjunct to dystrophin-restoring strategies (e.g., exon skipping or AAV-based gene therapy). Pilot combination studies to confirm additivity/synergy should precede a larger clinical trial.

This study has several limitations. First, we did not measure serum myostatin levels in aged *mdx* mice. In aged *mdx* mice, circulating myostatin is markedly reduced and can fall below the analytical sensitivity of standard ELISA assays, limiting reliable detection in advanced disease states (31, 45). In our cohort, pilot measurements frequently approached the assay floor, preventing robust between-group comparisons. Although this precluded direct confirmation of target suppression, the pharmacodynamic/functional signals—significantly elevated anti-myostatin IgG, lower serum CK, and improved rotarod performance—are consistent with pathway engagement. Future studies will incorporate higher-sensitivity platforms (e.g., enhanced ligand-binding/bridging assays, LC–MS, or aptamer-based assays) to verify circulating myostatin suppression in aged animals. In parallel, we will perform antibody characterization, including competitive binding/affinity testing, epitope and isotype profiling, and cell-based neutralization of myostatin-SMAD2/3 signaling, to more precisely relate humoral responses to biological activity. Notably, in young *mdx* mice, comparable anti-myostatin titers were accompanied by reductions in circulating myostatin (18), supporting the plausibility of target engagement in the current setting despite assay sensitivity constraints. Second, our assessment of muscle function relied on rotarod performance, which primarily reflects neuromuscular coordination rather than direct muscle strength. We did not include grip strength measurements, limb muscle weights, or fiber type–specific cross-sectional area (CSA) analysis, all of which are critical for more accurately evaluating changes in muscle mass and strength (43). Incorporating these endpoints in future studies will be essential to fully elucidate the therapeutic efficacy of BLS-M22. Third, the lack of detailed antibody characterization, such as binding affinity and neutralizing capacity, represents a significant limitation. While a significant increase in anti-myostatin IgG titers was observed, the absolute titers were modest, which may be partially due to age-associated immune senescence in the host. Future studies should incorporate affinity and functional binding assays to determine the neutralizing capacity of induced antibodies and optimize antigen design and delivery strategies to enhance immunogenicity.

In summary, an *L. casei* expressing a modified human myostatin protein successfully induced circulating anti-myostatin antibodies and conferred functional improvements in aged *mdx* mice, although its efficacy in advanced DMD appears limited without concomitant dystrophin restoration. As an oral, cost-effective, and immunologically distinct platform, BLS-M22 represents a novel approach with potential applications beyond DMD, including sarcopenia and other muscle-wasting conditions. Future studies should focus on optimizing dosing strategies, assessing long-term safety, and evaluating its efficacy relative to other myostatin-targeting modalities to clarify its broader therapeutic utility.

## Data Availability

The dataset generated and analyzed during this study is available in the Mendeley Data repository at Lee (44).
