# Supplementary figures and images for: Myostatin inhibition with orally administered Lactobacillus casei expressing a modified human myostatin protein: functional benefits and translational potential in advanced Duchenne muscular dystrophy
Source: Front Neurol. 2026 Jan 13;16:1693484. doi: 10.3389/fneur.2025.1693484 (PMC12836393; doi:10.3389/fneur.2025.1693484)

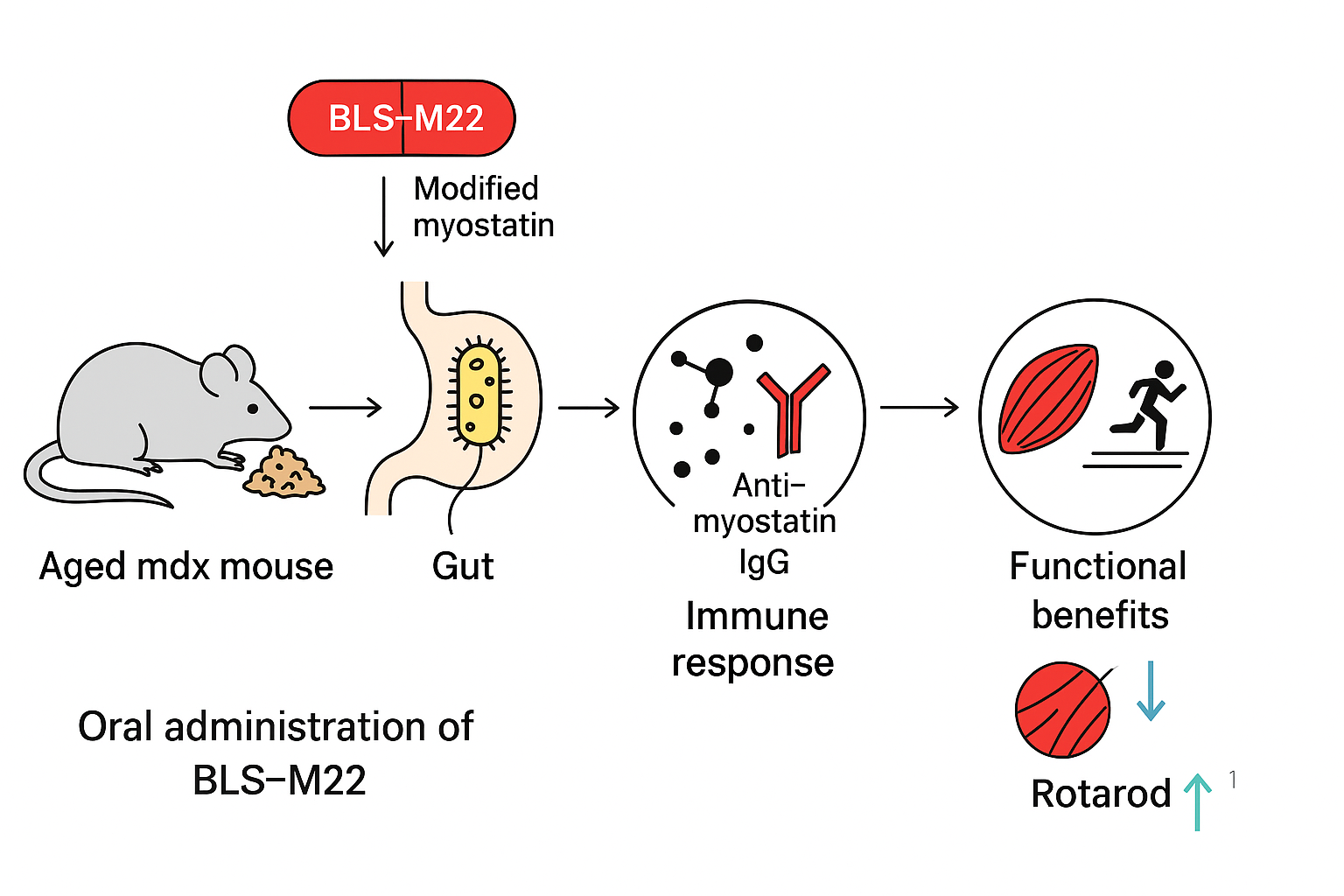

Supplement: Supplementary file 1 [file Image_1.TIFF]
